# Supplementary figures and images for: Risk factors of mortality in neonates with neonatal encephalopathy in a tertiary newborn care unit in Zimbabwe over a 12-month period
Source: PLOS Glob Public Health. 2022 Dec 20;2(12):e0000911. doi: 10.1371/journal.pgph.0000911 (PMC10021203; doi:10.1371/journal.pgph.0000911)

S1 Fig: Data flowchart


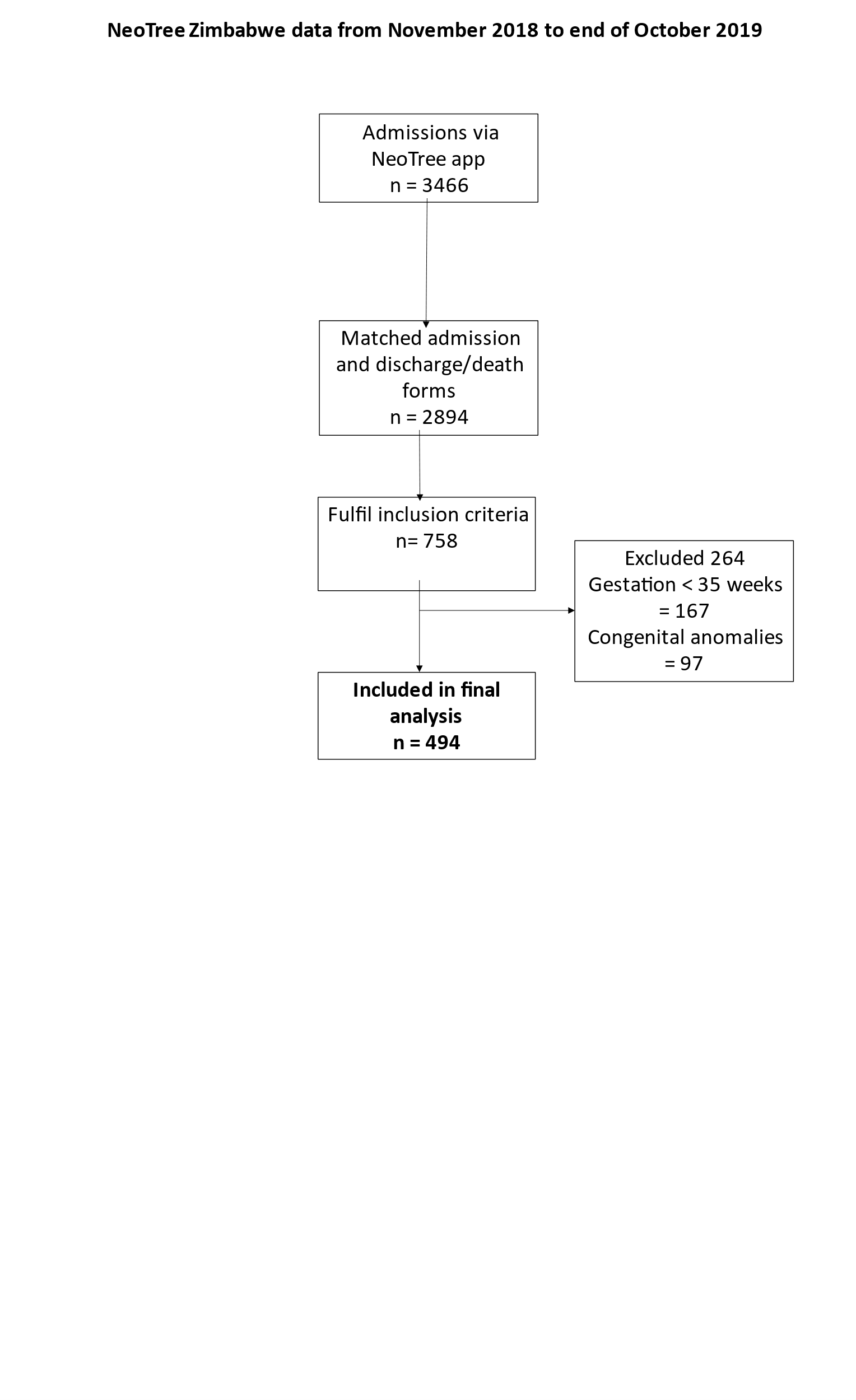

Supplement: S1 Fig — (DOCX) [file pgph.0000911.s001.docx]
