# Supplementary material for: Risk factors of mortality in neonates with neonatal encephalopathy in a tertiary newborn care unit in Zimbabwe over a 12-month period
Source: PLOS Glob Public Health. 2022 Dec 20;2(12):e0000911. doi: 10.1371/journal.pgph.0000911 (PMC10021203; doi:10.1371/journal.pgph.0000911)
Supplement: Attachment — Submitted filename: Response to Reviewers_Final.docx [file pgph.0000911.s003.docx]

Response to Reviewers

We are very grateful for the time taken by reviewers, and for the opportunity to revise and resubmit this article. Responses to reviewers’ comments are below.

1. *Please ensure that the funders and grant numbers match between the Financial Disclosure field and the Funding Information tab in your submission form. Note that the funders must be provided in the same order in both places as well.*

The Financial Disclosure field now reads: Funders include the Wellcome Trust: Digital Innovation Award (215742/Z/19/Z: PI: Heys) and the Healthcare Infection Society (SRG 201802004). Dr F. Fitzgerald is supported by the Academy of Medical Sciences and the funders of the Starter Grants for Clinical Lecturers scheme. This study and Drs M. Heys and F. Fitzgerald are further supported by the National Institute for Health Research, Great Ormond Street Hospital Biomedical Research Centre. The funders had no role in study design, data collection and analysis, or preparation of this report.

The Funding Information tab reads the same, but we are unable to add the detail about the Digital Innovation award. The grant numbers are matching in both places.

2. *Please update your online Competing Interests statement. If you have no competing interests to declare, please state: “The authors have declared that no competing interests exist.”*

The competing interests statement has now been amended to read: The authors have declared that no competing interests exist

3. *Please provide separate figure files in .tif or .eps format only and remove any figures embedded in your manuscript file. Please also ensure that all files are under our size limit of 10MB.*

The separate figure files have now been provided in .tif format, they are less than 10MB and there are no embedded figures in the manuscript.

4. *We have noticed that you have uploaded Supporting Information files, but you have not included a list of legends. Please add a full list of legends for your Supporting Information files after the references list.*

Supporting information legend has now been added to the manuscript after the References list. It now reads:

Supporting Information Legends:

**Supplementary Material 1: Data Flowchart**

**Supplementary Material 2: STROBE Checklist**

5. *Please review your reference list to ensure that it is complete and correct. If you have cited papers that have been retracted, please include the rationale for doing so in the manuscript text, or remove these references and replace them with relevant current references. Any changes to the reference list should be mentioned in the rebuttal letter that accompanies your revised manuscript. If you need to cite a retracted article, indicate the article’s retracted status in the References list and also include a citation and full reference for the retraction notice.*

The reference list has been checked and is complete and correct.

Additional Editor Comments:

1. *In the conclusion section of the abstract, the authors state, “The Thompson score, a validated, sensitive and specific tool for diagnosing neonates with NE was a good predictor of worse outcomes in this setting.”. To be able to call a characteristic as a good predictor one ought to evaluate its discrimination (AUROC, sensitivity, specificity etc), calibration and clinical usefulness analysis which were not performed in this article. It would be more appropriate to call Thompson score as an independent risk factor than a good predictor at this stage.*

This sentence has been amended to read: “The Thompson score, a validated, sensitive and specific tool for diagnosing neonates with NE was an appropriate predictive clinical scoring system to identify at risk neonates in this setting.”(line 58)

2. *The rationale behind exclusion criteria aren’t explained, particularly for the preterm < 35 weeks of gestation.*

Neonatal encephalopathy is defined as “a heterogeneous, clinically defined syndrome characterized by disturbed neurologic function in the earliest days of life in an infant born at or beyond 35 weeks of gestation, manifested by a reduced level of consciousness or seizures, often accompanied by difficulty with initiating and maintaining respiration, and by depression of tone and reflexes”. This is the established definition of NE and therefore used in the exclusion criteria^1^. The following sentence has been added: “as per the established definition of NE”. (line 183)

3. *In table-1, I see many continuous variables being categorized and univariate analysis has been performed. Why they were done so and not considered as continuous variables?*

The continuous variables were categorised as the banding of variables into groups made more sense for clinical interpretation. For example, Temperature <36.5^0^C is defined as “hypothermic”, 36.5-37.4^0^C is defined as “normal temperature” and >37.4^0^C defined as “hyperthermic”, rather than leaving as a continuous variable. For Thompson score Mild NE: score 1-10, Moderate NE: 11- 14 and Severe NE: 15-22. Gestation was banded into late pre-term, term and post-dates. Maternal age; adolescent mothers (<19 years old), advanced maternal age (geriatric pregnancy) (≥35 years old). The birthweight banding was selected according to the standardised low birth weight (LBW); <2500g, Very low birth weight (VLBW) <1500g, and then large for gestational age (LGA); >4000g. We did consider these variables as continuous variables and were aware of the risk of losing power, however with losing power we gained clinical interpretability.

4. *Please add STROBE checklist in the supplementary file.*

STROBE checklist added to supplementary files

Reviewer 1:

*Inclusion criteria “Diagnosis of Meconium aspiration syndrome AND associated convulsions” should be clarified. Does it mean infants with MAS are only included if they are observed to have convulsions? What if these infants have neurological signs and high Thompson score but were not observed to have seizures?*

We have amended the inclusion criteria to clarify. Infants with MAS were included if they were observed to have convulsions or abnormal neurological signs; defined as abnormal tone or activity in addition to exposure to meconium. If they only had respiratory symptoms, they were not included in the cohort. If the infant had a high Thompson score they would be included under the fourth inclusion criteria: “Thompson score >10 at admission (A Thompson score >10 was selected as cut off for moderate disease with reference to the original paper). Similarly if they had an Apgar score at five minutes <7 they were also included. (line 175-180)

*Risk factors of interest: Gender?? Do the authors mean sex? If not how was the assignment of gender determined at birth?*

We have amended this to sex. (line 188 and 241)

*Table 1: gestational age < 37 weeks does not accurately describe the study population. It should be revised to 35 – 37 weeks.*

Now amended. (line 241)

*Table 1: Risk factors for neonatal sepsis: What does “1 x risk factor” mean? Same style of reporting should be used in each section of the Table.*

This has been amended to read: “1 risk factor” for consistency

*Conclusions: The authors have not presented data to inform the conclusion that “Improvements in perinatal care could be achieved through data capture and quality improvement systems, such as the Neotree, that focus on adherence to best practice.” The role of Neotree in this study is not explicit.*

A short paragraph has been added to the discussion to explain this conclusion; “Data capture, analysis and ongoing informed quality improvement are essential for improving health services. The data used in this study, prospectively collected by the Neotree, is a rich resource of information which can be used by the local teams to inform ongoing quality improvement projects.” (line 392-395)

Reviewer 2:

*Title: Substitute risk with risk factors*

Amended in manuscript; RISK FACTORS OF MORTALITY IN NEONATES WITH NEONATAL ENCEPHALOPATHY IN A TERTIARY NEWBORN CARE UNIT IN ZIMBABWE OVER A 12-MONTH PERIOD

*Line 94- Please specify what is HRSs*

High resource setting, amended in the manuscript (line 100)

*Line 122- You mention lack of services. Would help to leverage some of this data to make a case a for provision of these services and highlight this in your discussion.*

Thank you for this comment. This has now been highlighted at the end of the discussion. “The data and results from this paper will be presented to the management board at SMCH and Ministry of Health and Child Care to leverage the need for the provision of essential drugs and resources for the supportive management of these NE neonates. Following the findings of this study we are in discussion with the Obstetric team in how best to implement quality improvement projects with the aim of improving the intrapartum care of these at-risk neonates.” (line 396-400)

*Regarding MICE- Did you include a summary measure of the entire Thompson scale, or also examined the missingness at both the item and summary score of Thompson? In making this decision, it may be helpful to examine the missingness at both the item and summary level. If there is very little item missingness within each scale (i.e. if some items for a scale are observed then they are all observed) and analysts will use only the summary scales, then imputing the summary scales may make sense. Graham (2009) suggests that creating and imputing a scale score is appropriate when at least half of the items are observed, the items have high coefficient alphas, and all of the item‐total correlations are similar. In contrast, if these conditions are not met it may make sense to impute the items themselves and then construct the summary scales using the observed and imputed data. (*[*https://www.ncbi.nlm.nih.gov/pmc/articles/PMC3074241/*](https://www.ncbi.nlm.nih.gov/pmc/articles/PMC3074241/)*)*

The Neotree was only capturing the Thompson summary score for this dataset, this had been manually calculated by the inputting health care professional. Therefore, we imputed the Thompson score’s final values and not its components. As the functionality of the Neotree has been adapted and improved over time the application is now able to calculate the score from the individual items, and the individual item scores are also captured within the Neotree dataset.

In a further paper reviewing Thompson score (currently in progress), using the Neotree data from 1^st^ January 2021 to 31^st^ January 2022, the Thompson score outcome was missing in 104 out of 590 (17.6%) included newborns. Of these 104 babies with missing data, 47 were missing only one component of the score with 57 with no information at all on Thompson score components. Scores were imputed for the 47 with only 1 component missing with the remaining 57 excluded. Therefore 9% were missing only 1 component, so we are confident that we are within the boundaries described in the paper by Graham (2009). We have added a sentence to the methods section to clarify this. (line 209-211)

*Line 310-Could the lower CFR be due to your explicit exclusion criteria?*

Our exclusion criteria included neonates <35 weeks and those with gastroschisis or severe congenital abnormalities. NE is not diagnosed in babies <35 weeks so that exclusion criteria cannot explain the lower CFR. Indeed, our clinical exclusion criteria are similar to the two comparative studies quoted in the discussion: Cavallin et al’s paper and Namusoke et al’s. The main difference is that we also excluded unmatched records as the data were incomplete for these neonates – it is possible that unmatched babies were more likely to have died (and that death of the baby would have been more likely to have led to incomplete recording of outcome and thus unmatched data). However, when reviewing the data for all discharge outcomes (including the unmatched cases) there was no real difference in CFR compared to the complete matched dataset.

*-Alluding the authors to this imp paper to consider, if relevant* [*https://www.ncbi.nlm.nih.gov/pmc/articles/PMC8075215/*](https://www.ncbi.nlm.nih.gov/pmc/articles/PMC8075215/)

Thank you for highlighting this paper, in this paper “outborn” NE neonates were found to be at highest risk. We did not find that in this review and this has now been included in the discussion. (line 347-350).

Reviewer 3:

*1. The authors mention in the abstract and elsewhere (page 2, page 3) that most deaths due to neonatal encephalopathy occur in sub-Saharan Africa. This is quite far from the truth, although the burden in that part of the world is high. Please refer to the article by Lawn et al, Lancet 2005 more carefully (reference 2 in your manuscript).*

Thank you for highlighting this point. We have now amended the abstract and the introduction to reflect this. Line 39-40 and Line 66-69

*2. The term "neonatal encephalopathy" is used throughout instead of hypoxic-ischemic encephalopathy (HIE), although the authors go on to describe therapeutic hypothermia as the intervention for this condition. Neonatal encephalopathy could be due to metabolic disorders, stroke and other conditions. If that term is used because of difficulty in diagnosing HIE specifically given the local context, please state that explicitly.*

The term “neonatal encephalopathy” was chosen due to the difficultly in accurately diagnosing HIE in this context. HIE can only be used if there is evidence of perinatal asphyxia as the primary cause of the encephalopathy, this cannot be used due to the difficulty in determining cause in this setting. We have now highlighted this in the paper. (line 70-72)

*3. The authors have described the Sarnat score as being EEG-dependent (page 4, line 100). Most trials (and many observational studies) involving infants with HIE in recent years have used a modified Sarnat score. It does not require an EEG.*

The original Sarnat scoring system does rely on EEG findings to make up part of the score. The modified version, as described in recent HIE papers does not rely on EEG but often the studies have included more advanced investigations, such as MRI or EEG, which are not readily available in LRS. The Thompson score in comparison was designed to be conducted with less training and less technical investigation availability. This has been amended in the text. (line 104-107)

*4. The meaning of the subheading "Inclusion criteria (to be met by 1 or more)" on page 7 is not clear.*

This has been clarified in the text, the individual inclusion criteria have now been numbered in the text. (line 172)

*5. Among the variables in the logistic regression model were activity (including lethargy/seizures), tone and Thompson score. It is worth noting that Thompson score itself incorporates these other variables. Did the authors note any multicollinearity?*

Thank you for this comment. We assessed collinearity in the models by chi-squared tests, which of course were significant with respect to tone and activity. We calculated the VIF (Variance inflation factor) using the R library car for the non-imputed dataset, and found that the generalised VIF (GVIF), adjusted by the complexity (measured in degrees of freedom), of the covariate for Thompson score was 1.148. Thus, the total variance in the model, assuming no collinearity, would be inflated by 14.8%, which indicated that these associations did not have an influential impact on the model’s power. We have added references to library car, and to a paper explaining GVIF’s, as well as these sentences to the paper; “Collinearity was assessed by Pearson’s χ^2^ and, using the R library car, generalised variance inflation factors adjusted for the degrees of freedom corresponding to each covariate(GVIFs).” (Lines 214-215), and “Regarding collinearity in the model, firstly chi-squared tests showed, as suspected, significant associations between categorised Thompson Score and tone and activity. However, GVIFs values indicated that these associations didn’t have an influential impact on the model’s power.” (Lines 307-311).

References:

1.	Executive summary: Neonatal encephalopathy and neurologic outcome, second edition. Report of the American College of Obstetricians and Gynecologists' Task Force on Neonatal Encephalopathy. *Obstet Gynecol* 2014; 123: 896-901. 2014/05/03. DOI: 10.1097/01.AOG.0000445580.65983.d2.
